# Human-AI interaction in safety-critical network infrastructures

**DOI:** 10.1016/j.isci.2025.113400

**Published:** 2025-08-20

**Authors:** Marco Mussi, Alberto Maria Metelli, Marcello Restelli, Gianvito Losapio, Ricardo J. Bessa, Daniel Boos, Clark Borst, Giulia Leto, Alberto Castagna, Ricardo Chavarriaga, Duarte Dias, Adrian Egli, Andrina Eisenegger, Yassine El Manyari, Anton Fuxjäger, Joaquim Geraldes, Samira Hamouche, Mohamed Hassouna, Bruno Lemetayer, Milad Leyli-Abadi, Roman Liessner, Jonas Lundberg, Antoine Marot, Maroua Meddeb, Viola Schiaffonati, Manuel Schneider, Thilo Stadelmann, Julia Usher, Herke Van Hoof, Jan Viebahn, Toni Waefler, Giacomo Zanotti

**Affiliations:** 1 Politecnico di Milano, Milan, Italy; 2 INESC TEC, Porto, Portugal; 3 SBB Swiss Federal Railways, Bern, Switzerland; 4 Delft University of Technology, Delft, the Netherlands; 5 EnliteAI, Wien, Austria; 6 Zurich University of Applied Sciences, Zurich, Switzerland; 7 University of Applied Sciences and Arts Northwestern Switzerland, Olten, Switzerland; 8 NAV Portugal, Lisboa, Portugal; 9 Fraunhofer IEE, Kassel, Germany; 10 University of Kassel, Kassel, Germany; 11 Reseau de Transport d’Electricite, Paris, France; 12 IRT SystemX, Paris, France; 13 Deutsche Bahn, Berlin, Germany; 14 Linköping University, Norrköping, Sweden; 15 Flatland Association, Bern, Switzerland; 16 University of Amsterdam, Amsterdam, the Netherlands; 17 TenneT, Arnhem, the Netherlands

**Keywords:** Artificial intelligence, Artificial intelligence applications, Human-computer interaction

## Abstract

Artificial Intelligence (AI) is transforming every aspect of modern society. It demonstrates a high potential to contribute to more flexible operations of safety-critical network infrastructures under deep transformation to tackle global challenges, such as climate change, energy transition, efficiency, and digital transformation, including increasing infrastructure resilience to natural and human-made hazards. The widespread adoption of AI creates the conditions for a new and inevitable interaction between humans and AI-based decision systems. In such a scenario, creating an ecosystem in which humans and AI interact healthily, where the roles and positions of both actors are well-defined, is a critical challenge for research and industry in the coming years. This perspective article outlines the challenges and requirements for effective human-AI interaction by taking an interdisciplinary point of view that merges computer science, decision-making sciences, psychological constructs, and industrial practices. The work focuses on three emblematic safety-critical scenarios from two different domains: energy (power grids) and mobility (railway networks and air traffic management).

## Graphical abstract

**Figure:**
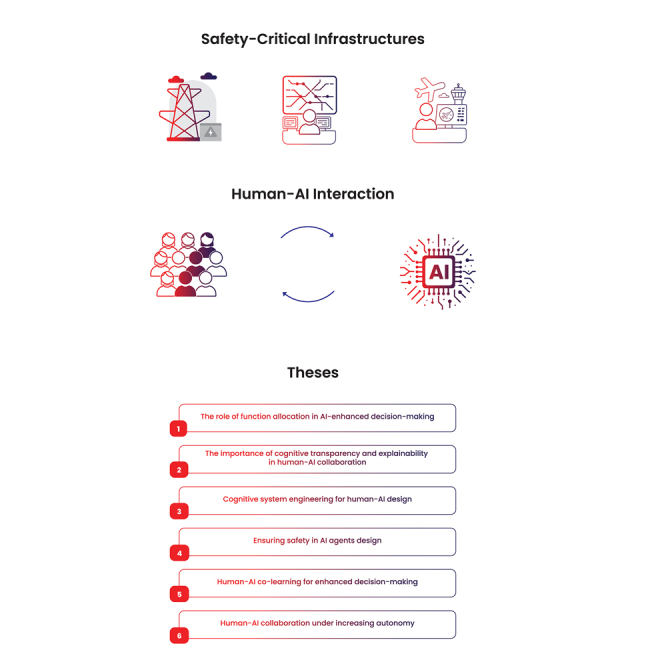

## Introduction

Artificial Intelligence (AI) has the potential to enhance the flexibility and resilience of safety-critical network infrastructures to address global challenges such as climate change impacts,^1^ facilitating the seamless integration of renewable energy sources,^2^ increasing demand from mobility and energy infrastructures, and optimizing resources/assets to postpone the need for significant capital investments in infrastructure reinforcement.^3^ Despite these advantages, AI faces several challenges. These include ensuring robustness, reliability, transparency, and ethical compliance to avoid issues such as errors and adversarial attacks. Additionally, AI must manage the complexity and uncertainty associated with aging assets and the non-stationarity introduced by increasing demand for energy and mobility networks. Finally, AI needs to address scalability limitations, particularly in methods such as reinforcement learning (RL), which struggle in large-scale network infrastructures.

The widespread adoption of AI is driving a new and inevitable interaction between humans and AI systems, particularly in processes that require real-time decision-making and forecasting. Traditionally, these infrastructures have been managed by humans relying on expertise, control, and supervision software at different levels of automation. Examples of that are air traffic management^4^ and power grid operations.^5^ In scenarios such as AI-assisted operations in power grid control rooms,^6^ such human-AI interactions are crucial. Although current AI technologies can incorporate human feedback, such as integrating human preferences in RL^7^^,^^8^^,^^9^ or facilitating interactive natural language conversations to explain AI models,^10^ they are not inherently designed to optimize the overall efficiency of socio-technical systems–hybrid systems composed of technical artifacts, human beings, institutions, and rules^11^–nor to maximize human performance and engagement consistently. This implies that current applications of AI cannot fully leverage this form of human-AI interaction, calling for new advancements in scientific research.

This paper integrates industry-specific knowledge from three safety-critical domains – power grid, railway network, and air traffic – where operational scenarios are typically characterized by multiple features that make the decision-making process particularly challenging. Indeed, such systems often consist of complex structures composed of multiple interconnected subsystems, requiring many decisions to be made within a limited amount of time. Furthermore, they are frequently affected by stochasticity, dynamic changes over time, and the need to handle cascading events and extreme cases. These characteristics not only make AI highly relevant in such environments but also reveal the limitations of current methods. Specifically, they highlight the need for AI systems that are not only robust and scalable but also designed to collaborate with humans in meaningful ways. As we will discuss in common decision-making aspects, despite domain-specific contexts, critical infrastructures face shared decision-making challenges, including complex human-AI interaction, multi-stakeholder coordination, and trade-offs under uncertainty, highlighting the need for a common conceptual framework.^12^

To succeed in these domains, AI must go beyond accuracy and performance—it must be trustworthy. Trustworthiness^13^ refers to a broad set of properties that capture both the technical and ethical dimensions of system design and use, including safety, robustness, transparency, fairness, interpretability, and explainability.^14^ These properties are critical for ensuring that AI systems can be accepted and relied upon in complex, high-stakes environments. This need is confirmed by the inclusion of trustworthiness requirements in the emerging regulation of AI systems, in particular, in the EU AI Act.^15^ As automation increasingly takes over cognitive tasks, systems must also preserve human skills, maintain human agency and oversight, and support effective interaction with AI.^16^ Achieving this requires transparent AI agents that help humans understand their outputs, learn from them, and assess their limitations.^17^

Complementing this technical perspective, recent frameworks such as “Meaningful Human Control”^18^ and “Human Readiness Levels”^19^ stress the importance of designing for effective human-AI collaboration. These approaches recognize that trust must be supported by systems that actively engage human decision-making, learning, and motivation.

In this context, joint decision-making between humans and AI can leverage the complementary strengths of both, ensuring that humans remain engaged and informed. Human learning in these settings involves developing an accurate understanding of the task and the AI’s behavior, supported by feedback and experimentation.^20^ Motivation to collaborate with AI depends on providing meaningful tasks, autonomy, and timely feedback. A promising direction to support this is *co-learning*,^21^ where humans and AI continuously learn from each other to improve overall performance. This requires AI agents to be autonomous yet collaborative, capable of adapting to humans and shared goals.^22^ While progress is being made, real-world examples of such systems remain limited. Therefore, this article offers a concise overview of practical use cases and requirements in three safety-critical infrastructures, highlighting key challenges and research directions (theses) to improve both AI capabilities and human-AI interaction.

## Decision-making in the power grid, the railway network, and the air traffic management

This section discusses the common challenges in decision-making across the three aforementioned safety-critical infrastructures (i.e., power grid, railway network, and air traffic), adopting a use case oriented approach that highlights the synergy between human expertise and AI-driven solutions. The goal is to identify cross-domain similarities in how decisions are made under uncertainty, time pressure, and system constraints, and, by aligning perspectives from different infrastructures, it contributes to establishing a foundational understanding of shared decision-making dynamics and to informing the design of joint human-AI decision systems.

### Common decision-making aspects

**Figure 1:**
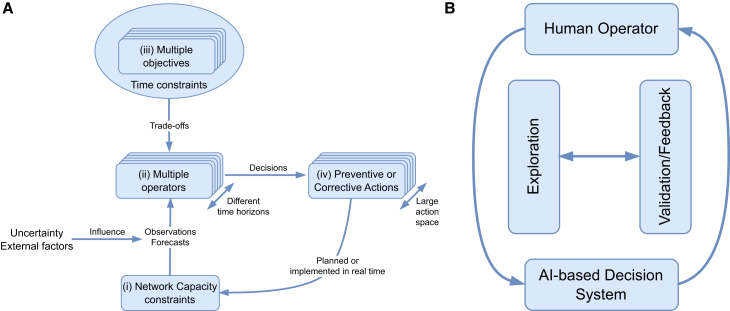
Decisions in safety-critical network infrastructure operations (A) Decision-making in safety-critical network infrastructure operations involves three key aspects: (i) managing network capacity constraints, identified via observations and forecasts of the network state, and influenced by uncertainty and external factors; (ii) involving multiple operators across different time horizons, ranging from long-term planning to real-time operations; (iii) operating under time constraints while balancing trade-offs between multiple objectives; and (iv) deciding on preventive or corrective actions selected from a large action space and planned or implemented in real time, respectively. (B) The decision-making process is iterative, involving exploration and validation tasks: Exploration assesses potential courses of action, while validation evaluates these actions against system objectives and constraints. This dynamic back-and-forth interaction integrates interconnected decisions, contributing to overall infrastructure management.

To identify common challenges in the decision-making process across all domains, scenarios were described and analyzed for each domain with industrial stakeholders in a joint workshop. These scenarios, which will be discussed in challenges in today’s operation and use cases section and extensively detailed in,^23^ are defined by involving representatives from power network operators (*Réseau de Transport d’Électricité* – RTE, TenneT), railway network operators (*Deutsche Bahn* – DB, *Schweizerische BundesBahnen* – SBB), and an air traffic management organization (NAV Portugal). In these scenarios, human operators face complex decision-making challenges that arise from a combination of external events, collaborative dynamics, conflicting objectives, and tight time constraints (Figure 1A). These decisions involve iterative interactions between human expertise and AI-driven insights, aiming to balance operational demands with system objectives (Figure 1B). Even if the decision context is different for each domain (which can be explained by the fact that each infrastructure remains intrinsically different), a high degree of similarity in the characteristics of the decision-making process was observed, specifically (see^23^ for a complete analysis).

(1) **Human-AI interaction.** Decision-making involves human operators and AI agents collaborating through manual, co-learning, and autonomous approaches, with iterative processes of exploration and feedback to refine and align actions with system objectives.
(2) **Multiple operators and other stakeholders.** Decisions involve coordination among diverse stakeholders (e.g., airport operators, power grid operators, train dispatchers) operating across various time frames, from long-term planning to real-time adjustments.
(3) **Action type.** Decision-making includes preventive or corrective actions, which can be planned or executed in real time. We distinguish between general actions taken by operators/AI and specific measures – concrete operational steps or plans addressing specific events.
(4) **Action space complexity.** The action space is large and comprises both discrete and continuous elements. Its complexity grows with system size, such as the number of power grid nodes, flights, or trains, making decision-making increasingly challenging.
(5) **Network capacity and external events.** Operators manage constraints resulting from disruptions, emergencies, or external factors such as maintenance activities and public events. These constraints are influenced by uncertain observations and forecasts, including weather conditions and human behavior variability.
(6) **Time resolution.** Real-time analysis enables immediate responses to urgent issues, while short-term analysis focuses on daily adjustments and preventive actions. Medium- to long-term analysis supports strategic planning and forecasting, preparing the infrastructure for future demands and challenges.
(7) **Trade-off analysis on conflicting objectives.** Operators must navigate trade-offs between competing objectives, such as meeting system needs while minimizing adverse impacts. Effective decision-making requires balancing these trade-offs, including weighing the probability and consequences of critical events to ensure safety and system integrity, or balancing operational demands with environmental goals such as reducing ${\text{CO}}_{2}$ emissions. Prioritizing tasks effectively is crucial for maintaining operational efficiency.

The following subsection provides examples of current practices and use cases that illustrate this decision-making process in action.

### Challenges in today’s operation and use cases

In today’s operations, power grid engineers are highly specialized, requiring detailed studies, accurate planning, and complex decision-making rather than merely following established protocols. They rely heavily on simulation tools with real-time and forecast data but have limited access to decision-support tools such as automated assistants.^6^^,^^24^ When addressing issues, engineers manually explore solutions and verify them using simulations. They can adjust grid connectivity, re-dispatch generation, limit consumption, or use battery storage to modify power flows, identifying the best actions for each specific context. Despite the range of options, their process depends on experience and manual simulation.^5^

An industry-driven AI use case proposes an AI assistant to support operators by recommending actions and strategies for real-time congestion management.^25^ The AI assistant should function bidirectionally, learning continuously from operator feedback, as illustrated in Figure 1B. This use case aligns with the schematic in Figure 1A. *Network capacity constraints* arise from thermal, voltage, and stability limits of power grid lines. Thermal limits depend on the maximum current a line can carry without exceeding its temperature rating, considering both instantaneous and short-duration thresholds. System-wide limits, such as voltage control, dynamic stability, and inertia, also restrict transfer capacity. Congestion occurs when these limits are exceeded, under both *N* (all elements available) and N−k (up to *k* outages, typically k=1) conditions. *Objectives* include managing overloads through remedial actions, maximizing renewable integration by reducing emergency redispatch of thermal units, and easing operator workload. The *trade-off* involves balancing the operational impact of an event, estimated via forecasts or real-time analysis, against its probability, usually derived from *ex ante* statistical studies of past events or forecasted by a statistical learning model. Depending on problem complexity, *multiple operators* may coordinate, such as control centers, field teams, market participants, or interconnected power grid operators. A lead operator is designated *ex ante* by operational rules (e.g., geographic responsibility or escalation to management). Key *observations* include the current grid state – measurements and topology (e.g., breaker positions) – used to assess loading and margin. Operators must also know the availability of actions, especially real-time flexibilities (e.g., cooldown times before switching). To *forecast* future conditions, inputs such as planned topological changes, generation and demand forecasts, maintenance schedules (with criticality), and electricity market signals are essential. *Uncertainty* can come from *external factors* such as storm or fire risks, major events (e.g., the Olympics), or incidents (e.g., accidents or protests) that may disrupt grid operations. Regarding *time constraints*, each decision, anticipatory or reactive, has a Last Time To Decide (LTTD), the latest point when action must begin for its effect to occur before the deadline. LTTD is computed by subtracting the action’s lead time from the deadline. For example, in response to an overload alarm, LTTD ensures intervention before thermal expansion forces an automatic line disconnection. Congestion management typically combines a) *preventive actions*, planned in advance when constraints are foreseeable, lead time is critical, or risk is high; and b) *remedial actions*, activated in real-time when fast-acting flexibilities are available. The choice depends on trade-offs involving availability, LTTD, cost, and effectiveness.

In railway network operations, densely planned schedules are frequently disrupted by unexpected events such as delays, infrastructure defects, or short-term maintenance. Maintaining smooth operations requires skilled personnel in control centers to monitor traffic flow around the clock and make quick re-scheduling decisions.^26^ These measures include adjusting a train’s speed, path, or platform. In densely used networks, local re-scheduling decisions can impact the entire traffic flow and propagate effects into the future, making this a complex decision-making task that integrates extensive context under *time* and *network capacity* constraints,^27^ aligning with the schematic in Figure 1A. *Network capacity constraints* are shaped by train frequency, scheduling density, and operational strategies such as prioritizing specific train types (e.g., high-speed or freight). As for *temporal constraints*, emergency situations such as accidents or technical failures often demand real-time responses, with decisions needed within minutes (*remedial actions*). Short-term operational adjustments (*preventive actions*), such as rerouting due to temporary obstructions or adapting to demand fluctuations, may allow slightly longer time horizons, typically from minutes to a few hours. The railway system requires the collaboration of *multiple operators*, encompassing those managing infrastructure, train operations, maintenance, and integration with other transport modes. This multiple environment is necessary to address the diverse constraints and ensure efficient, safe railway operations, particularly when integrating AI technologies. It is necessary to balance *trade-offs* for the punctuality of different trains, e.g., expanding capacity to accommodate more trains or passengers might strain resources or degrade service quality, affecting punctuality, comfort, and overall customer experience. *Uncertainty* arises from *external factors* such as unpredictable timing and duration of maintenance or upgrade projects (e.g., due to material shortages), extreme weather conditions requiring operational changes, and technical failures such as signal malfunctions or rolling stock breakdowns that cause unplanned delays and disruptions.

Railway network operators explore different modes of human-AI interaction and different degrees of automation to improve rescheduling performance. The different modes and degrees of automation are: a) highly automated AI re-scheduling systems that monitor the real-time state of trains and tracks, detect issues, decide automatically on actions, and execute them. Supervisors review system’s performance, adjusting parameters such as prioritization criteria, delay thresholds, or recalculation algorithms as needed; b) human-AI joint decision-making systems, where an AI assistant can support the exploration of alternative re-scheduling solutions or validate suggestions by human operations. Human operators can also validate alternative AI re-scheduling solutions based on operational priorities or additional contextual information not integrated into the AI system. Humans and AI continuously monitor the ongoing traffic and both decide on actions for rescheduling in a continuous exploration and validation loop (see Figure 1B).

Airspace sectorization divides airspace into manageable regions called sectors to ensure safe and efficient air traffic management by reducing controller workload and optimizing traffic flow.^28^ Currently, this task is solely managed by air traffic control supervisors, who decide when and how to split or merge sectors based on situational demands and available personnel.^29^ While scattered information is available across platforms, no integrated decision-support system is available to assist supervisors or automate the sectorization process, considering trajectory efficiency (e.g., flight time and fuel burn) and sector capacity limits. The long-term vision of the Single European Sky ATM Research (SESAR) program anticipates that tasks will eventually be performed collaboratively by hybrid human-AI teams.^30^ The industry AI-oriented use case features an AI-based system that monitors real-time ATM data, predicts sectorization needs, and implements plans either as recommendations or automatically.

Considering the schematic of Figure 1A, for this use case, *network capacity* is influenced by airspace dimensions, route structure, the availability of aeronautical systems and equipment, traffic demand, airport infrastructure, and staff availability. *Constraints* emerge from unpredictable events that reduce nominal sector capacity, such as military airspace activation, adverse weather, disruptive incidents, dynamic sectorization, and controller workload. Collaborative decision-making involves multiple stakeholders, including technical supervision and maintenance teams, air traffic controllers, airlines, airport operators, the EUROCONTROL network manager, and national air forces. Nonetheless, the final decision typically rests with a single operator, either a supervisor or a tactical air traffic controller. *Uncertainty* results from a range of *external factors*, such as partial airspace closures, operational disruptions (e.g., system failures, staff strikes, corrective maintenance), adverse weather, sector overloads, cybersecurity incidents, and in-flight emergencies. Some events, such as military airspace activation, known weather systems, scheduled maintenance of aeronautical systems, or anticipated staff shortages, can be forecasted in advance, enabling partial mitigation. Air traffic management requires balancing *multiple objectives*, e.g., a) *safety* vs. *capacity*, where increasing the number of aircraft in a sector or reducing separation may strain controller workload and increase the risk of critical events, or b) *flexibility* vs. *predictability*, where real-time adjustments (e.g., re-routing or trajectory changes) enhance responsiveness but reduce the predictability required for coordinated planning across the network. Regarding *temporal constraints* and *decisions*, the following categorization exists: a) *pre-tactical*, taken up to 1–2 h in advance, allowing planned measures such as re-sectorization in response to expected constraints (e.g., military airspace activation, balloon launches); and b) *tactical*, made within minutes to respond to real-time events such as sudden staff shortages (e.g., illness and fatigue), capacity overloads in adjacent sectors, emergencies, or last-minute activation of restricted airspace, and may involve measures such as flow adjustments, re-routing, or temporary changes to sector boundaries.

For this use case, AI provides visualized sector configurations on a map-like interface and learns from logged interactions with human supervisors, as depicted in Figure 1B. At lower automation levels, humans evaluate AI recommendations, request explanations, and adjust decisions. Higher automation levels range from “management by consent,” where AI acts with human approval, to full automation with human oversight limited to post-implementation revisions. In general, the role and feasibility of human oversight are still critical issues. While adequate human oversight is increasingly required by current regulations (e.g., AI Act), it should be acknowledged that the extent and way in which human oversight is actually feasible remains an open question.^31^^,^^32^

Human operators across these three infrastructures and use cases face a substantial cognitive load, as effectively managing and learning from these tasks requires considerable mental effort. This challenge, analyzed in^33^ for power grid control rooms under both normal and emergency conditions, results from the inherent complexity and fragmentation of the systems they oversee. Rather than increasing this burden, AI should aim to alleviate it by simplifying information processing, reducing the number of screens and tools human operators need to monitor, and providing contextual insights that enhance decision-making without overwhelming them. The reduction in cognitive load should not come at the cost of decreased transparency or control for human operators.

Finally, the Assessment List for Trustworthy Artificial Intelligence (ALTAI)^34^ was applied to perform an *ex ante* evaluation of these use cases across multiple dimensions, with emphasis on technical robustness and safety. This assessment (see^23^ for a detailed analysis) showed that AI-based decision systems in safety-critical contexts must be resilient to cyberattacks, data disruptions, and model uncertainties. Robustness metrics are essential during training and operation to detect adversarial inputs and compromised outputs. A human-in-the-loop design is essential to prevent critical failures, ensuring that final decisions remain under human supervision. Adaptability should be supported through transfer and time-adaptive learning, while continuous monitoring and stress testing help maintain reliability and reproducibility. Fault tolerance, technical reviews, and fallback mechanisms are required to manage uncertainty, including clear operator notifications and the ability to revert to manual control.

## Proposed design of enhanced human-AI systems

The interaction between humans and AI in safety-critical infrastructures presents a unique set of challenges that remain not completely addressed by existing frameworks. These challenges stem from the complex interplay of requirements for transparency, trust, and explainability, coupled with the necessity for robust and safe decision-making. Approaches that holistically integrate human and AI capabilities while addressing these concerns are notably uncommon (or even absent), leaving critical gaps in designing, deploying, and maintaining safe and effective systems.

### The AI side of human-AI interaction

*Black-box* AI models,^35^ while capable of achieving remarkable accuracy, hinder transparency and explainability, which are important for promoting trust in safety-critical contexts.^36^ Systems relying solely on such models often fail to meet the demands of safety-critical operations, where human operators must understand and trust AI decisions. In addition to human operators, other stakeholders (e.g., supervisors, managers, clients, and regulators) need to understand the models either to decide about their use or to retrospectively analyze their use (e.g., in case of an accident). To address this, AI agents must be designed with components that can be understood by humans, ensuring that the decision-making process aligns with human cognitive processes.

Transparency is a way to make understanding possible. In addition, it is an enabler for effective collaboration between humans and AI and for promoting trustworthy AI.^37^ Transparency should be integrated into the four stages of the human learning cycle: (*i*) during concrete experience, by explaining various factors of the process to encourage exploration; (ii) during reflective observation, by prompting reflection and hypothesis formulation about interrelated factors; (iii) during abstract conceptualization, by providing data-based evidence for or against the human’s hypotheses; and (iv) during active experimentation, by enabling safe real-world exploration and immediate feedback on outcomes. Furthermore, ensuring this property in safety-critical infrastructures requires capturing the characteristics of the corresponding decision-making processes and properly exploiting them. Indeed, instead of implementing large *black-box* models, one should exploit the known domain peculiarities of the use cases under analysis. Examples of that are the integration of *distributed*, *hierarchical*, or *knowledge-assisted* approaches in decision-making problems.

**Figure 2:**
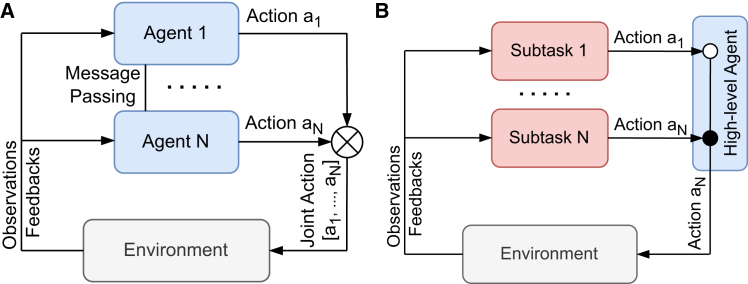
Comparison between distributed and hierarchical decision-making solutions (A) Distributed decision-making involves multiple agents, each observing (part of) the state of the system/environment. The agents may communicate with one another to exchange messages and individually decide on an action. The collective action applied to the system is the union of the actions chosen by all the agents. (B) Hierarchical decision-making involving a high-level agent overseeing the execution of the actions. This structure allows for a layered approach to decision-making, where subtasks handle low-level actions applied to the system/environment, and the high-level agent manages the overall strategy.

*Distributed* decision-making processes (Figure 2A) are methodologies where the responsibility for making decisions is divided among multiple decision points, each controlled by a different AI agent or subsystem.^38^^,^^39^^,^^40^ This approach allows for the decomposition of a complex global decision into a series of simpler, interconnected local decisions, which can also be better understood by human agents. By distributing the decision-making process, the system can leverage localized information, making it more adaptable, scalable, and resilient to changes or disruptions in specific areas.^41^ In complex systems based on a network structure, this paradigm is particularly advantageous. For instance, in a railway network, the system is typically divided into regions, each managed by a control center responsible for overseeing operations within its jurisdiction. These regional centers make decisions regarding train scheduling, maintenance, and conflict resolution for their specific area. However, the effectiveness of the overall railway system depends on how well these regional decisions are coordinated to ensure a seamless flow of trains, minimize delays, and maintain safety standards, and distributed methods were already applied to railway systems.^42^ This structure also aligns closely with the way power grids operate. In power grids, control centers are responsible for managing specific areas of the grid, such as balancing supply and demand, ensuring grid stability, and addressing faults in their areas. Similar to the railway network, decisions made at a local/regional level – such as topological changes to re-routing electricity flows – must be integrated, due to cascading effects, into a coherent global strategy to ensure the entire grid remains stable and efficient. Multi-agent RL has been applied to coordinate both active and reactive power control in photovoltaic generation systems within power grids.^43^ Open challenges in this field involve integrating the existing network structure, which includes control rooms and decision points functioning as decision-making nodes. These challenges encompass associating distributed AI agents with these control nodes and determining the optimal information sets for effective decision-making.^44^ Distributed approaches allow achieving transparency since the decision process carried out in each decision point is simpler and, for this reason, more interpretable and understandable by a human being.

*Hierarchical* decision-making solutions (Figure 2B) provide a structured approach to managing complex problems by breaking them down into high-level decisions and corresponding sequences of interconnected low-level actions.^45^^,^^46^ This *hierarchical* organization reflects the temporal and logical dependencies among decisions, allowing the system to handle complexity while maintaining clarity and understanding for human operators, who can better grasp the overall system goals.^47^ For instance, an operator in the power grid might receive a directive to “reduce the load in Region A by 20%,” along with an explanation of how the proposed low-level actions – such as activating local generators and rerouting surplus power – will contribute to achieving this goal. Hierarchical methods have been employed for optimal energy management and control of distributed energy resources in power grids.^48^ Similarly, in railway management, an operator might be advised to “alleviate congestion in Zone A by diverting trains to secondary routes,” with the AI providing a breakdown of which trains to reroute and when. Methodologies with hierarchical structures have been leveraged in railway networks.^49^ Open challenges include the development and analysis of effective *hierarchical* decision-making algorithms capable of scaling to complex continuous states and action spaces.^50^ Addressing these challenges is crucial for enabling the application of such approaches to large-scale critical infrastructures. Hierarchical methods promote transparency by clearly differentiating between high-level and low-level decisions, enforcing a more understandable view of the decision process.

*Knowledge-assisted AI* reduces the learning complexity by combining conventional planning approaches and human domain expertise with data-driven learning.^51^ Hybrid methodologies enable AI to focus on areas where human expertise is insufficient or incomplete while leveraging the strengths of established practices. For instance, integrating human-devised safety constraints into AI models can provide a foundation of reliability upon which learning-based improvements can build, ensuring that AI contributions align with predefined safety and operational goals. Knowledge-assisted methods, combining neural networks and symbolic structures, have been employed in aircraft collision avoidance systems.^52^ Open challenges include exploring less-studied representations, such as incorporating differential or algebraic equations directly into policies or value functions. Additionally, underexplored design patterns, such as leveraging symbolic methods as deliberative components within neural networks, present significant opportunities for advancement.^53^ Another challenge lies in developing modern approaches that integrate constraints directly into neural network architectures, analogous to^54^ but adapted for deep architectures.^55^ *Knowledge-assisted* approaches favor transparency since they integrate learning elements with human knowledge, which is typically more explainable.

### The human side of human-AI interaction

**Figure 3:**
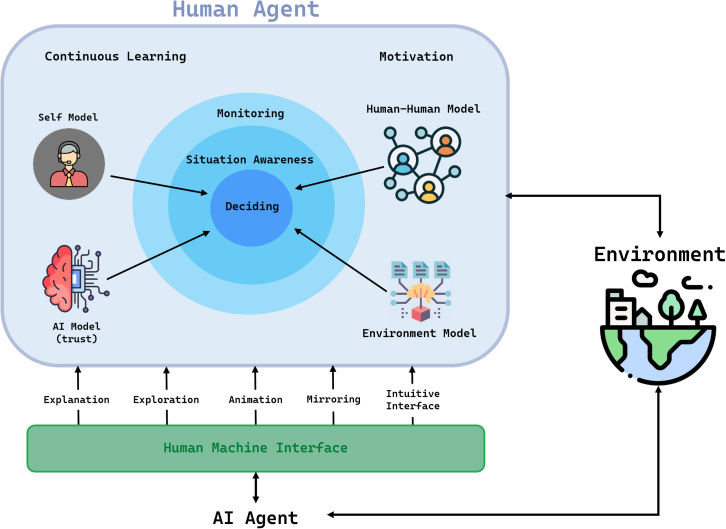
Model of human decision-making where AI can provide transparency to human-AI collaborative decision-making in the following forms (*i*) *explanation* (i.e., AI explains a subject matter), (ii) *exploration* (i.e., AI supports the human to explore/learn a subject matter), (iii) *animation* (i.e., AI animates the human to reflect on a subject matter), (iv) *mirroring* (i.e., AI mirrors individualized patterns in human behavior to make the human aware of their own biases and variabilities in decision-making), or (*v*) *intuitive interface design*. For effective and efficient decisions, AI must support *situation awareness* by assisting humans in *monitoring* networks and identifying critical points, enabling focused attention. This process relies on mental models encompassing representations of the *environment* (network knowledge), *human-human collaboration model* (understanding decision impacts on others), AI capabilities (*trust* and effective interaction), and *self-models* (awareness of decision patterns and biases). These models must be developed and *continuously* improved through AI-supported human *learning*. Moreover, AI should promote human *motivation* by complementing operators rather than overwhelming them.

Human decision-making is integral to the functioning of critical infrastructure. Consequently, AI needs to support corresponding macrocognitive processes (cf. Figure 3) such as monitoring and situation awareness.^56^^,^^57^ However, ideally, AI also supports learning, motivation, and trust to allow continuous improvement and to avoid over-reliance. In this context, human learning entails developing an *accurate mental model*^58^ of the AI, encompassing its capabilities, limitations, and behaviors. Such understanding enables operators to anticipate AI actions, interpret its outputs effectively, and collaborate seamlessly. Without a well-formed mental model, human performance may degrade, particularly in high-stress or dynamic scenarios.^59^ To achieve this, operators must continuously update their mental models.^60^ This involves incorporating new information and experiences,^61^^,^^62^ facilitating the dynamic learning process necessary for generating accurate mental representations of AI.

*Trust* is another pivotal factor in human-AI interaction. Trust in AI must align with the AI’s actual capabilities and scope of application.^17^ Mismatched trust levels, whether undertrust or overtrust, can lead to significant issues. Undertrust restricts the utilization of AI’s full potential, whereas overtrust – when human reliance on AI exceeds its reliability – can result in critical failures, which are particularly undesirable in safety-sensitive environments. For instance, during a power grid emergency, an operator placing excessive trust in AI recommendations might neglect manual interventions essential to mitigating a congested power line, potentially causing cascading failures and widespread outages. To support appropriate trust, AI agents must transparently communicate their capabilities and limitations. Simple explanations often fall short, as they require blind trust from users. Instead, AI agents should enable exploratory interactions, allowing users to investigate and refine their understanding of the system. This process leads to an informed trust grounded in experience and a thorough comprehension of the AI, thereby enhancing human-AI collaboration.

*Intrinsic motivation*^63^ is closely linked to an operator’s perception of the value and impact of their contributions. Without clear feedback on outcomes, operators risk disengagement, compromising the effectiveness of human-AI partnerships. Feedback mechanisms that clearly communicate the results of collaborative decisions are vital for maintaining motivation, promoting proactive behavior, and enabling calibrated trust.^17^ Both are critical in safety-critical systems, as they support the anticipation of future events.^64^ However, current AI decision-support systems often increase monotonous monitoring tasks, reducing user engagement and overstraining human capabilities.^16^^,^^65^ To address these challenges, AI design must integrate principles of intrinsic work motivation, ensuring that human operators retain an active and meaningful role in decision-making.

In human-AI collaboration, it is suggested that *function allocation* should not only rely on the humans’ abilities and performance. Rather, functions allocated to humans need to be perceived as meaningful.^66^ Consequently, not only the *what* and the *how* of task execution need to be addressed, but especially the *why*. Therefore, all interaction elements on the AI side, such as providing information or asking for information, must have a comprehensible purpose for the human. Furthermore, humans experience meaningfulness when the interrelations between their own activities and the activities of others (including the AI’s activities) are comprehensible and well-reasoned.

### Describing and designing human-AI interactions

For describing and designing human-AI interactions, lessons can be learned from human-automation interaction studies in Cognitive Systems Engineering (CSE). These studies do not focus exclusively on AI, but on any form of technology with which human operators need to collaborate. In cognitive engineering, the gist of human-automation teamwork is centered around a) team collaborations, with an emphasis on sharing and allocating control authority and autonomy between humans and automation, and b) automation transparency, aimed at providing deeper system insights for fostering understanding, trust, and acceptance.

Currently, a generic design “cookbook” for human-automation interaction does not (yet) exist. Instead, we advocate for the integration of two promising and related frameworks that can be used for both analyzing and designing human-automation interaction: Joint Control Framework (JCF)^4^ and Ecological Interface Design (EID).^67^ In its most succinct form, JCF focuses on team collaborations by describing the execution and planning of activities (e.g., sensing, deciding, and action implementation) when those are distributed over different agents. EID focuses more on achieving system transparency by visualizing the (physical and intentional) constraints on activities, which determine, in large part, the content, structure, and form of a human-machine interface. Integrating these two frameworks is possible due to their shared common ground, i.e., the CSE. CSE adopts an approach to human-machine interaction, where the design emphasis is first and foremost put on the work environment in which agents operate and activities take place. The work environment describes the boundaries for actions governed by physical laws, intentional principles, and processes. It essentially defines a safe envelope within which actions can take place, initially irrespective of who is executing the actions (e.g., humans or automated agents). At later (design and analysis) stages, agent-specific constraints are included (e.g., capabilities and limitations of both human operators and machines).

Given the shared CSE common ground, JCF’s emphasis on the execution and planning of activities (team collaborations), and EID’s focus on transparency by visualizing the constraints on activities, JCF and EID are complementary.^23^ EID visually reveals the constraints, relations, and action opportunities at all functional abstraction levels, and JCF modulates human-automation coordination on the activity level by putting (a sequence of) activities on a timeline describing on what abstraction level the system needs to be perceived, warranted by situational demands. In other words, EID prescribes what information should be portrayed and how, whereas JCF provides guidance on when to show information and how that links to specific activities (e.g., perceiving system information, formulating a decision, performing an action, among others).

## Perspectives

Building on the challenges and opportunities outlined in the previous sections, this part explores critical research directions for advancing human-AI collaboration in safety-critical environments. By addressing the interplay between human cognitive processes and AI capabilities, these directions aim to enhance transparency, trust, and mutual learning. Structured as six key theses, these perspectives provide a multidisciplinary framework to guide the development of human-AI systems that are not only effective and trustworthy but also adaptable to the complexities of real-world decision-making scenarios.

### The role of function allocation in AI enhanced decision-making

The integration of AI into safety-critical systems requires a deliberate and systematic allocation of functions between humans and AI. This allocation should optimize the strengths of both entities, achieving synergies that neither humans nor AI could accomplish independently. For example, AI excels at processing large datasets and identifying patterns, while humans bring contextual understanding, normative and ethical reasoning, and adaptability to novel situations. Function allocation should ensure that AI handles tasks requiring speed and precision while humans retain control over decisions requiring judgment, ethical considerations, and situation awareness. However, the functions assigned to the human must combine to form a psychologically sensible role, which is adequately supported by the AI. For example, it is a prerequisite for people to be committed to their role that they experience meaningfulness.^65^ Automation transparency must therefore ensure this and provide corresponding insights for the human.

#### The importance of cognitive transparency and explainability in human-AI collaboration

Human-AI collaboration extends beyond the explainability of AI to include mechanisms that enhance human cognitive processes. To achieve adequate situation awareness, humans need to monitor the network. This must also be supported by AI, for example, by helping humans identify critical points in the network so they can manage their attention accordingly. Cognitive transparency, which involves aligning AI outputs with human reasoning processes (described in Figure 3), is essential for effective collaboration. For instance, an AI agent assisting in controlling an air traffic system should not only present its conclusions but also explain the rationale behind them in a manner that aligns with the operator’s expertise and reasoning.

The aspects that are more about the mechanics of the situation can be explained to build up operator mental models, whereas the aspects that are dynamic and situation-dependent must be constantly renewed in a process of gaining situation awareness.^57^ We must thus distinguish between the explainer approach, which looks backward to motivate system activity, versus the transparency approach that shows the current status of the process.^56^ Considering the dynamics of network infrastructure operations, the temporal dimension is a key aspect. Taking time into account, the Construal Level Theory (CLT) framework departs from a normative perspective, considering aspects such as time available to make a decision versus the level of detail. At the extremes, there is the executive overview level (CLT 1) versus the detailed logs level (CLT 6).^68^ The CLT has been applied, e.g., to aviation.^69^ Turning toward cognition, decision-centric perspectives can be used to determine what needs explaining. Modeling then aims to describe a process of perceptions and actions surrounding a decision in critical episodes in more detail, on an event horizon. Abstraction here regards the external process, in terms of Levels of Autonomy in Cognitive Control (LACC). At the boundaries, to keep track of assets status or actions (LACC Level 1), versus to determine the situation and context (LACC level 6).^4^ The aspects that may require explaining are those that are not always shown to the operator, e.g., if a plan (level 3) is presented to an operator, then in an explanation, the relevant goals (level 5), any trade-offs (level 4), or implementation-based constraints (level 2) may be relevant to explain. For an operator to intervene or collaborate with an AI in control, transparency of these same aspects may instead be needed, perhaps with the means of adjusting the aspects. This transparency aims to promote trust, facilitate learning, and support motivation by enabling humans to understand, validate, and effectively interact with AI agents. However, empirical evidence on the impact of increased AI transparency on human performance (e.g., response time, workload, situation awareness) is limited and demands further research. Some exploratory studies advocate the use of hierarchical information presentation, such as “progressive disclosure,”^70^ to deliver explanatory information in a phased manner to avoid cognitive overload and display clutter.

#### Cognitive system engineering for human-AI design

Effective human-AI collaboration requires the application of cognitive system engineering principles to model decision-making processes and define system requirements. These models should account for human cognitive capacities and limitations, ensuring that AI agents are designed to complement, rather than overwhelm, human operators. Methods such as Ecological Interface Design^67^^,^^71^ and the Joint Control Framework^4^ are particularly valuable for modeling decision-making processes and defining requirements for function allocation and visual elements portrayed on an interface that align with human cognitive processes, including decision making, learning, trust, and motivation. For example, in railway network management, designing an AI agent to assist with train scheduling and traffic control involves understanding how operators process information and make decisions under time pressure. By aligning the AI agent’s functionalities with these cognitive processes – such as prioritizing trains based on their schedules and managing potential conflicts at junctions – engineers can significantly enhance systems’ safety, efficiency, and reliability.

Beyond the discussion of task and interface requirements, a more fundamental aspect of human-AI teaming is determining the extent to which human operators can and should understand machine-generated recommendations and actions. Stakeholder perspectives play a crucial role in shaping this understanding. For instance, tactical operators are typically not computer scientists and may neither need nor be expected to grasp the underlying algorithms, provided that the AI’s actions ensure safety. In contrast, policymakers and technical personnel may require deeper insights to assess how specific algorithm configurations and trained policies affect overall system performance. Ongoing debates within the AI community – particularly between advocates of interpretability versus explainability^35^ – highlight the lack of consensus on what humans should understand about AI systems and how that understanding should be achieved. Holzinger and Muller^72^ propose the concept of *causability* as an alternative, and potentially better, way of determining to what extent humans can understand a given machine explanation.

#### Ensuring safety in AI agents design

Safety is a primary concern in the design of AI agents for safety-critical environments. These systems must incorporate robust risk management mechanisms and provide quantitative guarantees of minimal performance, particularly in rare or corner-case scenarios. Such guarantees are essential to mitigating the negative perception of AI errors and promoting human trust in the system’s reliability. For instance, in power grid management, AI agents must undergo rigorous testing to address scenarios such as sudden power surges, equipment failures, or unexpected demand fluctuation. By ensuring that the AI can reliably recommend actions, such as rerouting power or isolating affected sections of the grid, even under extreme conditions, operators can trust the system to maintain network stability and safety. For railway systems, the system design ensures that no safety-critical situations can occur for rescheduling decisions because the signalization is independent of the human-AI decision-making system. The adoption of ethics-by-design approaches that allow the identification and management of trustworthiness-related properties (including safety) of the system since early stages of development is fundamental. This means methodologies for the early identification of functional and non-functional requirements and key performance indicators that are explicitly linked to trustworthiness,^12^ as well as suitable strategies for continuous risk management.^73^^,^^74^ Importantly, while the development of AI-specific processes for achieving and verifying regulatory compliance may be necessary, it is crucial that these processes are consistent with domain-specific methods and standards adopted by the operators and users of critical infrastructures.

#### Human-AI co-learning for enhanced decision-making

Human-AI co-learning involves a dynamic process where humans and AI evolve through mutual interaction. In safety-critical network control, these mental models involve representations of the environment (knowledge about the network to be controlled), human-human collaboration (understanding how one’s decisions impact others managing other areas of the network), representations of AI (understanding its capabilities and limitations to build trust and interact effectively), and representations of oneself (awareness of decision-making patterns and biases). Mental models must be developed and continuously improved through a human learning process supported by AI. This collaboration enables humans to refine their skills and understanding, while AI adapts its models based on human feedback. In safety-critical systems, co-learning enhances decision-making by leveraging the complementary strengths of humans and AI. For example, in air traffic management, AI can process vast amounts of real-time data from multiple sensors to optimize flight paths and prevent potential conflicts. Meanwhile, human air traffic controllers provide contextual knowledge, situation awareness, and normative judgment to address complex or unforeseen scenarios. This iterative process builds trust, improves performance, and enables safe and efficient airspace management that neither humans nor AI could achieve independently.

#### Human-AI collaboration under increasing autonomy

Increased automation and even full autonomy may be desirable for certain tasks. However, when full automation is not possible, i.e., when the human still needs to take a critical role in operations, it remains essential to integrate human needs into the design process. Research in human factors has been instrumental in addressing the limitations associated with assigning humans the role of a passive supervisory agent. In this role, human vigilance decreases quickly while fatigue increases. Furthermore, skills not used over longer periods are being lost. From this standpoint, for having the human-in-the-loop, it is a prerequisite to assign them an active role. One possible way to do so is to assign the human the role of a “director,” interacting with and giving directions to one or more AI agents, which in turn can manage and allocate subtasks either hierarchically or in a distributed fashion. It is argued that this fulfills human factors’ foundational requirements for interested human engagement as it supports an adequate human autonomy and situation awareness, which is not given in a standard supervisory role. In essence, the key to cultivating appropriate trust lies in designing AI systems that are not only advanced in their technical capabilities but also in their ability to engage with humans, promoting transparency, exploration, and feedback about performance and error boundaries. Such an approach ensures that trust in AI-based tools is informed by direct experience and a comprehensive understanding of AI’s error boundaries, leading to more effective and nuanced human-AI collaborations.

## The role of AI-friendly digital environments

To develop and benchmark novel human-AI systems, AI-friendly simulation environments – designed to support seamless AI integration, training, and interaction while replicating realistic operating scenarios of critical infrastructures – are essential. Examples of such open-source environments include: a) *Grid2Op*, which enables the development and evaluation of power grid operations agents^24^; b) *Flatland* for developing and testing solutions to train rescheduling problems^27^; and c) *BlueSky* for the validation of AI-driven solutions in realistic air traffic management scenarios using open data.^75^

Leveraging these digital environments allows organizations to promote internal AI innovation through in-house AI communities while facilitating collaboration and co-development with external AI networks.^76^ This approach promotes a cultural shift toward data sharing and collaborative construction of digital platforms for human-AI development and testing. It also promotes transforming traditionally rigid critical infrastructure business models into dynamic networks that integrate technological platforms, mobility and energy providers, and end-users, potentially as Testing and Experimentation Facilities (TEFs) for AI.^76^ Moreover, these efforts help address emerging legal and ethical challenges, including liability issues, which are particularly relevant given that energy and mobility are classified as high-risk sectors under the EU AI Act.^15^ These environments can also improve human operators’ training efficiency and effectiveness, especially when new technologies such as AI are available to support decisions.^77^

**Figure 4:**
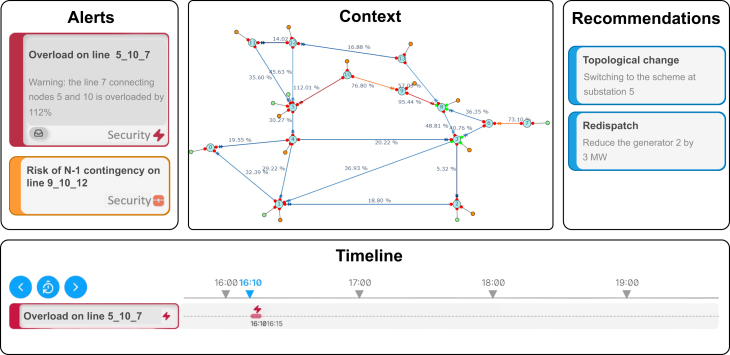
Experimentation of bi-directional virtual assistants for joint decision-making using the CAB framework’s prototype and its instantiation for the power grid use case It consists of four key panels: the *Context Panel*, displaying a real-time view of the environment (e.g., the power grid and its components) with tools such as zooming; the *Timeline Panel*, tracking time steps and event history for analysis; the *Alerts Panel*, listing notifications about risks and events (e.g., power line overloads and contingency risks); and the *Recommendations Panel*, offering AI-based suggestions that operators can adopt based on their expertise and the complexity of the situation (e.g., topological or redispatching actions with KPIs on the impact).

The development trajectory should aim for maximum generality by creating a multi-domain environment that integrates domain-specific digital environments while offering a suite of generic functionalities applicable across a vast majority of domains and use cases. These functionalities should include network and data representation, interaction mechanisms between controllers and simulations, user-system interfaces, training of learning controllers, evaluation tools, and support for reproducibility. Moreover, another research direction is toward creating experimentation capabilities of bi-directional virtual assistants for joint decision making. This will provide the opportunity to evaluate the forms of exchange between the human expert and an AI that continuously learns from the received information flows and the decisions made by humans, e.g., how to visualize the status, how to describe the explanations, and how to interact with the interface. A notable example in this direction is the Cockpit and Bidirectional Assistant (CAB) project, which created an open-source prototype^78^ with four key panels (a simplified representation is provided in Figure 4), which is being further enhanced within the AI4REALNET European project^79^ and available to various industrial applications such as power grids, railway networks and air traffic management. This prototype allows monitoring and evaluating the interactions between human operators and an AI that continuously learns from both incoming information flows and human decisions.^80^ The explanatory aspect of the AI’s recommendations is also central, adding value to the operator’s decision-making process. This has the potential to change the normal operation of an AI agent that supports decision-making by making a direct link between the human and the AI, making use of the operator’s implicit information to enhance its empathy with the operator. For instance, by using psychophysiological data from the operator^81^ and personalized models to retrieve in real-time the estimation levels of stress and cognitive performance, AI assistants will be able to make decisions not only based on the operational context, but also taking into account the status of the human operator, adapting the complexity, information and interaction of tasks with the user. We advocate this implicit symbiosis as a research line focused on creating AI assistants that look friendlier without the human explicit perception. In the future, virtual assistants will be able to determine the profile of the operator and their level of cognitive workload and adapt the information flows uploaded to the operator to manage a complex and/or atypical situation in the best conditions.

## Conclusions

For AI-based decision systems operating critical infrastructures, this work claims that more focus should be placed on optimizing the degree of decision support of AI to humans, aiming at achieving the best possible interaction between humans and AI (rather than simply deploying AI-based systems). To accomplish this, the goal should be to align system design with human cognitive processes and limitations and incorporate rigorous safety protocols rather than merely implement automation or AI. This vision of human-AI interaction not only addresses technical challenges but also offers an opportunity to redefine the role of AI as a collaborative partner in safeguarding critical systems.

In this vision, the explainability of AI is crucial for developing an accurate mental model, as it clarifies the AI’s decision-making process. However, it alone does not ensure effective human learning. Therefore, transparency is fundamental to understanding AI and provides clear, real-time insights into an AI’s activities, which is essential for profitable interaction in dynamic contexts. However, AI decision support can also go beyond the provision of comprehensible suggestions. This is the case when AI specifically supports cognitive processes of human decision-making (e.g., the ability of humans to develop situation awareness, recognize problems, or identify leverage points) with the aim of continuously increasing corresponding human capabilities. In this way, humans and AI act as a joint cognitive system and create true hybrid intelligence, leveraging the strengths of both humans and AI.

## Acknowledgments

The research leading to this work is part of the AI4REALNET (*AI for REAL-world NETwork operation*) project, which received funding from the European Union’s Horizon Europe Research and Innovation programme under the Grant Agreement No 101119527, and from the Swiss State Secretariat for Education, Research and Innovation (SERI). This project is funded by the European Union and SERI. Views and opinions expressed are however those of the author(s) only and do not necessarily reflect those of the European Union and SERI. Neither the European Union nor the granting authority can be held responsible for them.

## Author contributions

Conceptualization, M.M., A.M.M., M.R., G.L., R.J.B., D.B., C.B., A.C., R.C., D.D., A.Eg., A.Ei., Y.M., A.F., J.G., S.H., M.H., B.L., M.L-A, R.L., J.L., A.M., M.Med., V.S., M.S., T.S., J.U., H.V.H, J.V., T.W., and G. Z.; methodology, M.M., A.M.M., M.R., G.L., R.J.B., D.B., C.B., A.C., R.C., D.D., A.Eg., A.Ei., Y.M., A.F., J.G., S.H., M.H., B.L., M.L-A, R.L., J.L., A.M., M.Med., V.S., M.S., T.S., J.U., H.V.H, J.V., T.W., and G. Z.; investigation, M.M., A.M.M., M.R., R.J.B., D.B., C.B., A.C., R.C., S.H., M.H., M.L-A, J.L., A.M., M.Med., V.S., M.S., J.U., H.V.H, T.W., and G. Z; writing-original draft, M.M., A.M.M., M.R., R.J.B., C.B., S.H., M.L-A, A.M., M.S., J.U., and T.W.; writing-review and editing, M.M., A.M.M., M.R., R.J.B., D.B., C.B., A.C., R.C., Y.M., A.F., S.H., B.L., M.L-A, R.L., A.M., M.Med., M.S., T.S., J.U., H.V.H, J.V., and T.W.; funding acquisition, R.J.B.; supervision, M.M. and R.J.B.

## Declaration of interests

The authors declare no competing interests.
